# Elevated plasma levels of TIMP-3 are associated with a higher risk of acute respiratory distress syndrome and death following severe isolated traumatic brain injury

**DOI:** 10.1136/tsaco-2018-000171

**Published:** 2018-06-27

**Authors:** Carolyn M Hendrickson, Stuart L Gibb, Byron Y Miyazawa, Sheila M Keating, Erin Ross, Amanda S Conroy, Carolyn S Calfee, Shibani Pati, Mitchell J Cohen

**Affiliations:** 1 Division of Pulmonary and Critical Care Medicine, Department of Medicine, University of California San Francisco, San Francisco, California, USA; 2 Department of Laboratory Medicine, University of California San Francisco, San Francisco, California, USA; 3 Blood Systems Research Institute, San Francisco, California, USA; 4 Department of Surgery, University of California San Francisco, Zuckerberg San Francisco General Hospital, San Francisco, California, USA; 5 Department of Surgery, University of Colorado, Denver, Colorado, USA

**Keywords:** ARDS, TBI

## Abstract

**Background:**

Complications after injury, such as acute respiratory distress syndrome (ARDS), are common after traumatic brain injury (TBI) and associated with poor clinical outcomes. The mechanisms driving non-neurologic organ dysfunction after TBI are not well understood. Tissue inhibitor of matrix metalloproteinase-3 (TIMP-3) is a regulator of matrix metalloproteinase activity, inflammation, and vascular permeability, and hence has plausibility as a biomarker for the systemic response to TBI.

**Methods:**

In a retrospective study of 182 patients with severe isolated TBI, we measured TIMP-3 in plasma obtained on emergency department arrival. We used non-parametric tests and logistic regression analyses to test the association of TIMP-3 with the incidence of ARDS within 8 days of admission and in-hospital mortality.

**Results:**

TIMP-3 was significantly higher among subjects who developed ARDS compared with those who did not (median 2810 pg/mL vs. 2260 pg/mL, p=0.008), and significantly higher among subjects who died than among those who survived to discharge (median 2960 pg/mL vs. 2080 pg/mL, p<0.001). In an unadjusted logistic regression model, for each SD increase in plasma TIMP-3, the odds of ARDS increased significantly, OR 1.5 (95% CI 1.1 to 2.1). This association was only attenuated in multivariate models, OR 1.4 (95% CI 1.0 to 2.0). In an unadjusted logistic regression model, for each SD increase in plasma TIMP-3, the odds of death increased significantly, OR 1.7 (95% CI 1.2 to 2.3). The magnitude of this association was greater in a multivariate model adjusted for markers of injury severity, OR 1.9 (95% CI 1.2 to 2.8).

**Discussion:**

TIMP-3 may play an important role in the biology of the systemic response to brain injury in humans. Along with clinical and demographic data, early measurements of plasma biomarkers such as TIMP-3 may help identify patients at higher risk of ARDS and death after severe isolated TBI.

**Level of evidence:**

III.

## Introduction

Each year approximately 1.7 million people suffer a traumatic brain injury (TBI) in the USA, of whom 50 000 die and greater than 800 000 are left with some form of permanent disability.^1^ Beyond the primary neurologic injury, two-thirds of patients with TBI have extracranial complications that contribute to their deaths, and pulmonary complications are common among patients with severe TBI.^2–5^ Acute respiratory distress syndrome (ARDS) is a form of severe, acute lung injury characterized by increased vascular permeability resulting in protein-rich edema fluid flooding the alveolar space. The development of ARDS after TBI is associated with worse patient outcomes and higher healthcare costs.^6–10^ Understanding the molecular pathways involved in the systemic response to brain injury could lead to targeted therapies or improvements in supportive care after injury. Furthermore, the discovery of a biomarker profile that identifies patients with TBI who are most likely to benefit from novel therapies to prevent and treat ARDS could enhance study design and enrollment in future intervention trials.

Biomarker studies in observational cohorts of patients with TBI are needed to determine the clinical relevance and biological mechanisms of vascular leak in murine models of TBI. One such biomarker of interest is tissue inhibitor of matrix metalloproteinase-3 (TIMP-3). TIMP proteins interact with matrix metalloproteinases (MMPs) to regulate the extracellular matrix composition and play a key role in tissue remodeling and repair throughout the body.^11 12^ TIMP-3 is an appealing biomarker candidate for ARDS after TBI because it has been demonstrated to attenuate TBI-induced blood–brain barrier (BBB) permeability and is also abundant in the lung tissue.^13–16^ TIMP-3 stabilizes the vascular endothelium and blocks the vascular endothelial growth factor-A (VEGF-A) signaling by directly binding to VEGF receptor 2.^17^ Importantly, in animal models, TIMP-3 has been shown to promote normal endothelial barrier function,^14^ inhibit vascular endothelial permeability after injury,^13 18^ and regulate matrix degradation in the airspaces.^13^ Moreover, there are data to suggest that TIMP-3 is involved in neutrophil recruitment and regulation of inflammation in mouse models of acute lung injury.^19^ More recently, in rodent models of TBI treated with mesenchymal stem cells (MSCs) after injury, TIMP-3 was found to be a key soluble factor secreted by MSCs that mediated the protective effects of MSCs on the BBB. High doses of recombinant TIMP-3, administered acutely after TBI, attenuated BBB compromise, reduced neuroinflammation, and reduced cerebral edema.^18 20^

Although TIMP-3 appears to play an important role in cerebral edema after TBI, the association of TIMP-3 with ARDS after TBI has not been studied. Given murine studies demonstrating that TIMP-3 is a critical mediator of vascular endothelial barrier function in the brain and the lungs, we hypothesized that plasma levels of TIMP-3 may be altered and may be associated with ARDS and mortality after severe TBI. We tested this hypothesis by measuring TIMP-3 concentration in plasma obtained immediately after severe isolated TBI in subjects enrolled in a prospective cohort study of severely injured trauma patients.

## Patients and methods

### Patients

We retrospectively studied 182 patients with isolated severe TBI who were enrolled in a larger, prospective, observational cohort study at Zuckerberg San Francisco General Hospital between 2005 and 2014. Adult subjects meeting the criteria for the highest level trauma activation are eligible for enrollment in the larger cohort study. All subjects included in this retrospective study required mechanical ventilation and survived at least 6 hours from time of admission. Subjects suffered severe isolated TBI defined by an Abbreviated Injury Scale (AIS) Head score ≥3 with confirmed findings of TBI on head CT scans. Subjects were excluded if there were other serious injuries defined as AIS score ≥3 in any other body region. Plasma samples were not available for 30 of the 212 subjects (14%) who otherwise met the study inclusion criteria. Eleven healthy volunteers donated blood samples for TIMP-3 measurements.

### Sample collection and TIMP-3 measurements

The method of sample collection has been described previously in detail.^21^ Briefly, a 10 mL sample of blood was drawn within 10 minutes of arrival in the emergency department (ED). The median time elapsed from emergency medical services (EMS) dispatch to ED blood draw was 34 minutes in this cohort (IQR 28–48 minutes). The samples were immediately centrifuged and the plasma was stored at −80°C. Blood samples from healthy volunteers were processed and stored in a similar fashion. TIMP-3 levels were measured in duplicate using Milliplex MAP Human TIMP Magnetic Bead Panel 2 - Immunology Multiplex Assay (EMD Millipore) on the Luminex platform using a Labscan 200 analyzer (Luminex, Austin, Texas) and Bio-Plex manager V.6.1 software (Bio-Rad, Hercules, California). A 5-point logistic regression curve was used to calculate the concentration from the fluorescence intensity of the bead measurements. The standards ranged from 97 pg/mL to 100 000 pg/mL. During the 3-year study period, the intra-assay reproducibility was 5% coefficient of variablity and the interplate variability at the range of sample detection was 15%. Samples that were below the level of detection were assigned half the lowest detectable value for that analyte. The mean value of the two measurements was used for data analysis. Three extreme outlier values identified on the first round of measurements were measured a second time in duplicate, and the mean of the four assay results was used for data analysis.

### Data collection and outcome measures

Data were collected prospectively on patient demographics, mechanism of injury and severity, and subsequent hospital course. Patients were monitored until hospital discharge or death. As previously described, a rigorous two-physician ARDS adjudication protocol was used to identify cases according to the Berlin definition in the first 8 days of admission.^22 23^ Two physician investigators reviewed all chest radiographs from the first 8 days of admission for each patient with at least one PaO_2_ in the arterial blood to fraction of inspired oxygen ratio (PaO_2_: FiO_2_) of <300 and adjudicated ARDS cases by consensus. A monitoring board of clinicians in the hospital’s Division of Infectious Diseases identified patients with ventilator-associated pneumonia (VAP) using clinical, radiographic, and microbiologic data.

### Statistical analysis

Data are presented as mean±SD, median (IQR), or n (percentage). Univariate comparisons were made using Student’s t-test for normally distributed data and Wilcoxon rank-sum for skewed data. A non-parametric test for trend across ordered groups was used to test the association between the TIMP-3 quartile and the incidence of ARDS and mortality rates. In exploratory univariate analyses, α<0.2 was considered significant for further investigation with regression models. In all other analyses, α<0.05 was considered significant. All models were repeated after excluding the extreme outlier value for one subject with plasma TIMP-3 concentration of 19 450 pg/mL.

Multivariate logistic regression models were used to adjust for potential confounding and to illustrate that TIMP-3 contains additional predictive information compared with other markers of severity of injury or worse clinical outcomes (age, AIS Head score, arrival Glasgow Coma Scale (GCS) score, transfusion of blood products, vasopressor use, and shock identified by arrival base deficit, a measurement of a decrease in the total concentration of blood buffer base). Multivariable logistic regression models were tested with appropriate model checking. Initial models included race, sex, age, body mass index, AIS in the head and chest categories, crystalloid administration, base deficit, vasopressor use, massive transfusion (>10 units of packed red blood cells in 24 hours), and transfusion or individual blood products in the first 12 hours. These variables were selected a priori based on biological plausibility. Age was included as a dichotomous variable to satisfy linearity assumptions of the regression models. Individuals in the highest quartile of age, >60 years old, were compared with younger subjects. Difference of fitted beta coefficient (DFBETA) statistics were calculated to check for influential points. The influence of outliers was assessed by comparing the regression model results including all data with the regression models that excluded outliers identified by DFBETA statistics. Some covariates were serially eliminated using likelihood ratio testing. Multivariable logistic regression model fit was checked with the link test and Hosmer-Lemeshow test. Because TIMP-3 is not a commonly measured biomarker or clinically available laboratory value, it was scaled using the SD (2090 pg/mL) of the TIMP-3 measurements from the entire cohort excluding the extreme outlier of 19 450 pg/mL. To evaluate for possible misclassification of the 16 subjects who underwent massive transfusion and therefore may have had more significant injuries beyond TBI, we performed a chart review. The bleeding among the subjects who required massive transfusion was attributable to a combination of gunshot wounds (GSW) to the head, associated vascular, facial, or scalp injuries (including lacerations and degloving), and/or large estimated blood loss related to coagulopathy that developed during craniotomy procedures. Only one subject may have had a more significant injury in another body region that may not have been captured by the AIS scoring, a GSW to the clavicle and shoulder. To address limitations of the retrospective data analysis and concerns about misclassification of patients with more significant injuries beyond TBI, a sensitivity analysis of the multivariate models was performed, excluding 16 (9%) of 182 subjects who received massive transfusion. To address the possible influence of at-risk time and censorship by death on the association between TIMP-3 and ARDS, we used regression models with an exposure parameter by creating a variable indicating at-risk time. These models include time at risk as an offset variable. All analyses were performed using STATA V.13.

## Results

### Demographic and clinical features of the cohort

The median age of the subjects in this cohort with was 42 years (IQR 27–60 years). The distribution of race was Asian (20%), black (18%), Pacific Islander (1%), and white (60%), and race was not specified for 2% of the subjects. The cohort was 20% Latino. Subjects were predominantly male (74%) and most suffered blunt injuries (85%). Head injury in this cohort was severe; 157 (86%) subjects had an AIS Head score >3, 156 (86%) had a GCS score <14 on ED arrival, and 122 (67%) had a GCS score ≤8 on ED arrival. Overall mortality was 39% and the incidence of ARDS in the first 8 days of hospitalization was 27%. In this cohort, mortality rates did not differ between those who developed ARDS and those who did not (46% vs. 36%, p=0.23). Race and ethnicity did not differ by ARDS or vital status at discharge. Compared with subjects who did not develop ARDS, subjects with ARDS were more likely to have shock defined by a base deficit of <−4 on ED arrival arterial blood gas (51% vs. 29%, p=0.004) and to be treated with vasopressors on the day of admission (73% vs. 54%, p=0.003) (table 1). There was a trend toward more severe head injury (AIS Head score >3) among those who developed ARDS (94% vs. 83%, p=0.062). There was a trend toward a higher proportion of male subjects among those who developed ARDS compared with those who did not (84% vs. 70%, p=0.052). Compared with subjects who survived to discharge, subjects who died were older (mean age 53±24 vs. 40±18, p<0.001), had lower admission GCS score (median 3 vs. 8, p<0.001), were more likely to have a more severe head injury (AIS Head score >3) (99% vs. 78%, p<0.001), and were more likely to have shock defined by a base deficit <−4 on ED arrival arterial blood gas, systolic blood pressure <90 mm Hg in the first 12 hours after ED arrival, or treated with vasopressors on the day of admission (table 1).

**Table 1:** Demographic and clinical features of subjects with isolated severe traumatic brain injury by ARDS outcome and vital status at discharge

Patient characteristics	Without ARDS(n=132)	With ARDS(n=50)	P values*	Alive(n=111)	Dead(n=71)	P values*
Age (years)	46±22	44±20	0.78	40±18	53±24	**<0.001**
Male sex	92 (70)	42 (84)	0.051	85 (77)	49 (69)	0.26
BMI	25 (22–29)	26 (23–28)	0.54	25 (23–29)	25 (22–28)	0.56
Blunt injury	111 (84)	44 (88)	0.51	99 (89)	56 (79)	0.056
SBP <90 mm Hg in the first 12 hours	12 (9)	4 (8)	0.82	5 (5)	11 (15)	**0.011**
Any vasopressor on admit day	66 (54)	32 (73)	**0.031**	49 (48)	49 (77)	**<0.001**
Admit base deficit <−4	38 (29)	26 (52)	**0.003**	32 (29)	32 (45)	**0.025**
ED arrival GCS score	7 (3–10.5)	6 (3–10)	0.84	8 (4–13)	3 (3–7)	**<0.001**
Head AIS	5 (4–5)	5 (5–5)	**0.033**	5 (4–5)	5 (5–5)	**<** **0.001**
Head AIS score >3	110 (83)	47 (94)	0.062	87 (78)	70 (99)	**<** **0.001**
Craniotomy or craniectomy	44 (33)	24 (48)	0.068	37 (33)	31 (44)	0.16
Any chest injury (AIS score >0)	17 (13)	7 (14)	0.84	11 (10)	13 (18)	0.10
VAP†	5 (3)	7 (14)	**0.038**	16 (14)	5 (7)	0.13
TIMP-3 (pg/mL)‡	2260 (970–3460)	2810 (1820–4380)	**0.008**	2080 (950–3090)	2960 (1850–4650)	**<** **0.001**

Findings presented as n (%), mean±SD, and median (IQR) as appropriate.

Statistically significant results are shown in bold.

* P values refer to unpaired t-test for age, Wilcoxon rank-sum test for all other continuous variables, and unordered χ^2^ tests for dichotomous predictors.

† Patients with VAP identified before ARDS or in the first 8 days for subjects who did not develop ARDS.

‡ Plasma sample obtained on arrival to the ED.

AIS, Abbreviated Injury Scale or; ARDS, acute respiratory distress syndrome; BMI, body mass index; ED, emergency department; GCS, Glasgow Coma Scale; SBP, systolic blood pressure; TIMP-3, tissue inhibitor ofmatrix metalloproteinase 3; VAP, ventilator-associated pneumonia.

### Plasma TIMP-3 measurements

**Figure 1:**
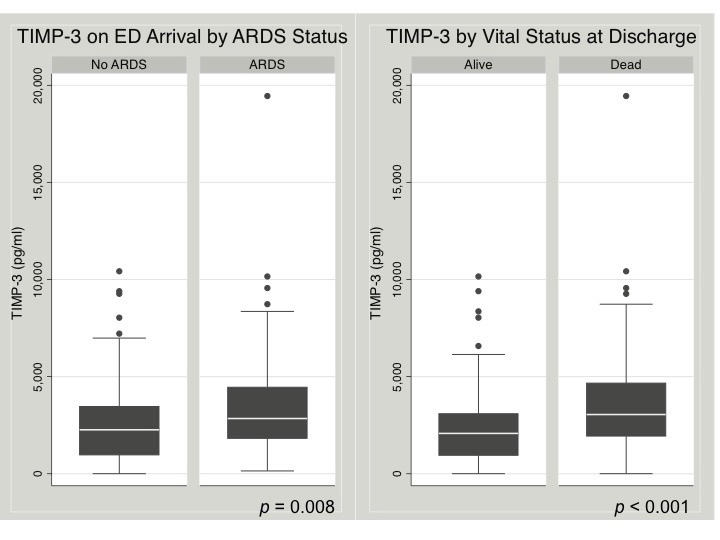
Early plasma tissue inhibitor of matrix metalloproteinase-3 (TIMP-3) levels are higher among subjects who developed acute respiratory distress syndrome (ARDS) and among subjects who died compared with those who did not. Box plots of plasma TIMP-3 levels on emergency department (ED) arrival by ARDS status (left) and vital status (right). P values refer to Wilcoxon rank-sum tests.

**Figure 2:**
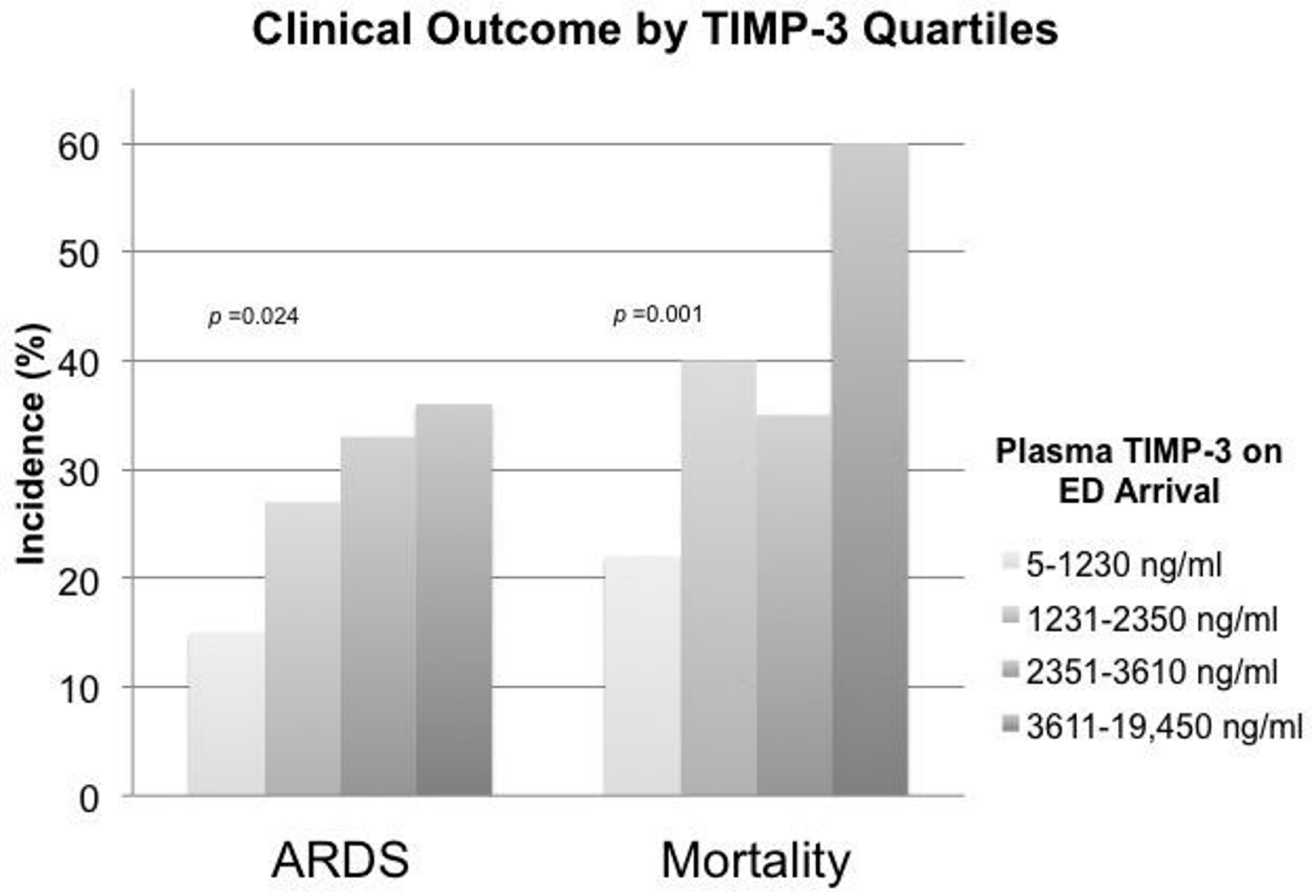
Higher incidence of acute respiratory distress syndrome (ARDS) and mortality rate among subjects with higher levels of plasma tissue inhibitor of matrix metalloproteinase-3 (TIMP-3) levels. There is a significant trend of ARDS incidence and mortality rates across increasing quartiles of TIMP-3. The results of non-parametric tests for trend across ordered groups are indicated by the p values. ED, emergency department.

The range of TIMP-3 among 11 healthy subjects was 20 pg/mL to 3700 pg/mL, with a median of 290 pg/mL (IQR 70–810 pg/mL). The range of TIMP-3 among 182 subjects with severe TBI was 5 pg/mL to 19 450 pg/mL, with a median of 2350 pg/mL (IQR 1230–3610 pg/mL) (table 2). TIMP-3 measured on ED arrival was significantly higher among patients who subsequently developed ARDS compared with those who did not develop ARDS in the first 8 days of admission (median 2810 pg/mL vs. 2260 pg/mL, p=0.008) (table 1, figure 1). Among subjects who survived beyond the first week after injury, TIMP-3 on ED arrival was significantly higher among those who developed ARDS compared with those who did not (median 2540 pg/mL vs. 1860 pg/mL, p=0.033). The incidence of ARDS significantly increased across TIMP-3 quartiles (non-parametric test for trend across ordered groups, p=0.013) (figure 2). TIMP-3 measured on ED arrival was significantly higher among patients who died compared with those who survived to discharge (median 2960 pg/mL vs. 2080 pg/mL, p<0.001) (table 1, figure 1). Compared with subjects who survived, TIMP-3 on ED arrival was significantly higher among subjects who died after 24 hours (median 3030 pg/mL vs. 2080 pg/mL, p=0.0005) and among subjects who died after 48 hours (median 3410 pg/mL vs. 2080 pg/mL, p=0.0007). These differences remained significant when the extreme outlier value of measured TIMP-3 was excluded from the analyses. The mortality rate significantly increased across TIMP-3 quartiles (non-parametric test for trend across ordered groups, p=0.001) (figure 2).

**Table 2:** Plasma TIMP-3* in healthy volunteers and subjects after traumatic injury

Clinical phenotype	TIMP-3 (pg/mL)
Healthy volunteers (n=11)	290 (70–810)
Isolated traumatic brain injury* (n=182)	2350 (1230–3610)

Findings presented as mean (IQR).

* Plasma sample obtained on arrival to the emergency department.

TIMP-3, tissue inhibitor of matrix metalloproteinase 3.

### Logistic regression models

Analysis with regression models showed that higher concentrations of plasma TIMP-3 on ED arrival were significantly associated with ARDS after severe isolated TBI. For each SD increase in plasma TIMP-3 measured on ED arrival, the unadjusted odds of ARDS increased significantly, OR 1.5 (95% CI 1.1 to 2.1, p=0.006) (table 3). This association was only mildly attenuated in models adjusted for important covariates: severity of head injury, base deficit, male sex, crystalloid infusion, and early transfusion of blood products, OR 1.4 (95% CI 1.0 to 2.0, p=0.036) (tables 3 and 4). The magnitude of association was unchanged in sensitivity analyses excluding patients who received massive transfusion, OR 1.5 (95% CI 1.0 to 2.3, p=0.033), and excluding patients who developed VAP before ARDS, OR 1.5 (95% CI 1.0 to 2.3, p=0.027) (table 3). Similarly, this association was not attenuated after adjusting for additional covariates associated with severity of illness including vasopressor use and GCS score on ED arrival (table 4).^24–26^ Furthermore, models that accounted for follow-up time did not substantively change the association between TIMP-3 and ARDS.

**Table 3:** Association of TIMP-3* and clinical outcomes after traumatic brain injury in univariate and full logistic regression models

Independent variables in model	For each SD change in TIMP-3OR (95% CI)†	P values
	**ARDS**	
TIMP-3	1.5 (1.1 to 2.1)	0.006
TIMP-3, AIS Head score, age>60 years, male sex, crystalloid*, platelet*, pRBC*, plasma* transfusion, shock by arrival base deficit	1.4 (1.0 to 2.0)	0.036
Sensitivity analysis: multivariate model‡ excluding subjects requiring massive transfusion§	1.5 (1.0 to 2.3)	0.033
Sensitivity analysis: multivariate model‡ excluding subjects who developed VAP before ARDS	1.5 (1.0 to 2.2)	0.036
	**Death**	
TIMP-3	1.7 (1.2 to 2.3)	0.002
TIMP-3, AIS Head score, age>60 years, male sex, crystalloid*, platelet *, blood*, plasma* transfusion, shock by arrival base deficit§	1.9 (1.2 to 2.8)	0.002
Sensitivity analysis: multivariate model‡ excluding subjects requiring massive transfusion	1.9 (1.2 to 3.1)	0.005

Crystalloid infusion included as a continuous variable indicating liters transfused in the first 12 hours of admission.

* Plasma sample obtained on arrival to the ED.

† Regression model output for scaled predictor variable is interpreted as the change in the OR for outcome of interest for each SD (2090 pg/mL) increase in the plasma concentration of TIMP-3 on ED arrival, holding all other variables in the model constant. AIS, models exclude subjects with AIS Head score=6 (non-survivable injury). Platelet, pRBC, and plasma (fresh-frozen plasma) coded as binary variables indicating any transfusion of the specific products in the first 12 hours of admission. Crystalloid infusion included as a continuous variable indicating liters transfused in the first 12 hours of admission.

‡ Multivariate model refers to full model with independent variables specified in the row above. Massive transfusion defined as requiring >10 units of pRBCs in 24 hours. Sensitivity analyses excluded 16 (9%) of 182 patients in the full cohort who required massive transfusion.

§ Base deficit of <−4 on ED arrival arterial blood gas analysis.

AIS, Abbreviated Injury Scale; ARDS, acute respiratory distress syndrome; ED, emergency department; pRBCs, packed red blood cells; TIMP-3, tissue inhibitor of matrix metalloproteinase 3; VAP, ventilator-associated pneumonia.

**Table 4:** Association of TIMP-3* and clinical outcomes after traumatic brain injury in univariate and multivariate logistic regression models

Independent variables in model	For each 1000 pg/mL increase in TIMP-3OR (95% CI)†	P values
	**ARDS**	
TIMP-3	1.5 (1.1 to 2.1)	0.006
TIMP-3, shock by arrival base deficit‡	1.4 (1.0 to 1.9)	0.031
TIMP-3, vasopressor on day of admission	1.6 (1.2 to 2.2)	0.004
TIMP-3, age>60	1.5 (1.1 to 2.1)	0.005
TIMP-3, GCS score	1.5 (1.1 to 2.1)	0.006
TIMP-3, AIS Head score	1.5 (1.1 to 2.0)	0.009
TIMP-3, platelet transfusion§	1.5 (1.1 to 2.1)	0.006
TIMP-3, pRBC transfusion§	1.5 (1.1 to 2.1)	0.007
TIMP-3, plasma transfusion§	1.6 (1.1 to 2.1)	0.005
TIMP-3, AIS Head score, male sex, age>60, crystalloid, platelet, blood, plasma transfusion§, shock‡	1.4 (1.0 to 2.0)	0.036
	**Death**	
TIMP-3	1.7 (1.2 to 2.3)	0.002
TIMP-3, shock by arrival base deficit‡	1.6 (1.2 to 2.2)	0.005
TIMP-3, vasopressor on day of admission	1.7 (1.2 to 2.4)	0.002
TIMP-3, age>60 years	1.7 (1.2 to 2.3)	0.002
TIMP-3, GCS score	1.6 (1.2 to 2.3)	0.005
TIMP-3, AIS Head score	1.7 (1.2 to 2.5)	0.001
TIMP-3, platelet transfusion§	1.7 (1.2 to 2.3)	0.002
TIMP-3, pRBC transfusion§	1.7 (1.2 to 2.3)	0.002
TIMP-3, plasma transfusion§	1.7 (1.3 to 2.4)	0.001
TIMP-3, AIS Head score, age>60 years, crystalloid, platelet, blood, plasma transfusion§, shock‡	1.9 (1.2 to 2.8)	0.002

Models exclude subjects with AIS Head score=6 (non-survivable injury).

* Plasma sample obtained on arrival to the ED.

† Regression model output for scaled predictor variable is interpreted as the change in the OR for outcome of interest for each 1000 pg/mL increase in the plasma concentration of TIMP-3 on ED arrival, holding all other variables in the model constant.

‡ Base deficit of <−4 on ED arrival arterial blood gas analysis.

§ Adjusted models control for transfusion of blood products in the first 12 hours of admission.

AIS, Abbreviated Injury Scale; ARDS, acute respiratory distress syndrome; ED, emergency department; GCS, Glasgow Coma Scale score on ED arrival; pRBCs, packed red blood cells; TIMP-3, tissue inhibitor of matrix metalloproteinase 3.

Analysis with regression models showed that higher concentrations of plasma TIMP-3 on ED arrival were significantly associated with death after severe isolated TBI. For each SD increase in plasma TIMP-3, the unadjusted odds of death increased significantly, OR 1.7 (95% CI 1.2 to 2.3, p=0.002) (table 3). The magnitude of this association was increased in a multivariate model adjusted for important covariates: severity of head injury, age, crystalloid infusion, early transfusion of blood products, base deficit, and in a sensitivity analysis of the multivariate model excluding patients who received massive transfusion (table 3). The association was not attenuated in several bivariate models adjusting for other markers of injury severity, such as vasopressor use and GCS score on ED arrival (table 4). In an unadjusted logistic regression analysis, subjects with the highest quartile of TIMP-3 concentrations, greater than 3610 pg/mL on ED arrival, had significantly greater odds of death. Specifically, the OR for death was 3.1 (95% CI 1.5 to 6.1, p=0.002) among subjects in the highest quartile of plasma TIMP-3 concentration compared with subjects in the lower three quartiles of plasma TIMP-3 concentration. Including TIMP-3 in the full multivariate logistic regression models with clinical and demographic characteristics improved the accuracy of predicting the outcomes of ARDS and death after TBI (area under the receiver operator curve (AUROC) 0.74 vs. 0.72 and 0.83 vs. 0.79, respectively).

## Discussion

In this observational cohort study, we have shown for the first time that among patients with severe isolated TBI, plasma TIMP-3 levels measured on ED arrival are associated with a higher risk of ARDS and a higher risk of death. This association was not attenuated in multivariate logistic regression models that controlled for covariates such as severity of head injury, vasopressor use, shock, volume of crystalloid infused, and transfusion of blood products. Importantly, higher levels of plasma TIMP-3 on ED arrival were associated with death occurring later than 24 hours and 48 hours from the time of admission. The association of TIMP-3 levels with outcomes at these later time points suggests that elevated TIMP-3 may identify early differences in the pathophysiologic response to injury that have durable effects on patient outcomes beyond identifying patients who are so severely injured they die shortly after presentation. Collectively, these findings suggest that TIMP-3 is a potentially important biomarker for predicting ARDS and death after severe TBI. The biological role, if any, of elevated TIMP-3 levels on arrival in the ED is unknown and requires further investigation.

Although complications after injury such as ARDS are common after TBI, mechanisms mediating these effects are not well understood. Although there is a growing body of evidence from animal models describing the molecular and physiologic mechanisms underpinning the brain–lung crosstalk after TBI, little is known about these mechanisms in humans.^27–35^ Identifying biomarkers for ARDS after isolated TBI is a relatively novel line of investigation. Most biomarker studies in ARDS exclude patients with TBI or do not distinguish isolated TBI as a distinct clinical phenotype within the general trauma population. As in other at-risk populations, it is likely that multiple biological pathways drive the pathophysiology of ARDS after TBI.^36–39^ Recently, in a cohort study of 200 patients with severe TBI, Aisiku *et al*^40^ found an association between ARDS and an elevation in early inflammatory plasma cytokines, interleukin (IL)-6 and IL-8, and anti-inflammatory cytokine, IL-10. Our study builds on these findings by testing the association of a biomarker of vascular stability with ARDS after TBI. The association between TIMP-3 and worse clinical outcomes after isolated TBI supports the hypothesis that the vascular endothelium has an important biological role in the pathogenesis of complications after injury, such as ARDS and death.

Identifying patients at the highest risk for ARDS after TBI would be clinically useful. For example, those at the highest risk for ARDS might benefit from the implementation of early lung protective ventilation protocols, closer monitoring for oxygenation deficits, or screening for evidence of cardiopulmonary volume overload with directed bedside ultrasound in the intensive care unit. Given an increased interest in the prevention and early treatments for acute lung injury, the development of reliable methods for identifying patients at the highest risk of ARDS is an important line of future investigations.^41 42^

Although TIMP-3 has not been studied in ARDS, it plays an important role in the mouse models of postinjury brain edema, and its role in vascular leak and localization in the lung tissue supports the biological plausibility for a role in ARDS.^18 20^ The association between TIMP-3 and poor clinical outcomes after isolated TBI is consistent with the hypothesis that dysregulated MMP activity may be involved in vascular endothelial stability and play an important role in the pathogenesis of complications after injury, such as ARDS and death, in patients with TBI. It is notable that the direction of the association between higher TIMP-3 levels and worse outcomes was unexpected. Since TIMP-3 administration has been shown to have a therapeutic effect in animal models of TBI,^18 20^ it is interesting that in this clinical study we found that elevated levels of TIMP-3 after TBI are associated with poor outcomes. We speculate that the differences between our findings and previously published data from mouse models may be explained by one of three hypotheses: (1) increased plasma TIMP-3 could be a surrogate marker for injury severity through either passive release of TIMP-3 from injured tissue or from an active homeostatic mechanisms to maintain the MMP:TIMP balance; (2) increased plasma levels of TIMP-3 may represent movement of endogenous and beneficial TIMP-3 activity into the systemic vascular compartment, away from the injured brain and lung tissue, impeding recovery from injury in these organs; and (3) the plasma levels of TIMP-3 in the patients are far lower than the therapeutic range tested in mice. The levels of TIMP-3 found in the plasma of injured patients (median of 2350 pg/mL) are 400-fold lower than the amount used for therapeutic purposes in preclinical models.^18 20^ It is also possible that TIMP-3 biology is fundamentally different between humans and mice.

Our study has some limitations. This is a retrospective, single-center study and may have limited generalizability. Larger follow-up studies would be needed to validate the use of TIMP-3 as a biomarker for ARDS and mortality after TBI. Although the AIS and GCS are widely used in trauma research, AIS Head and GCS scores do not capture much information about the nature of brain injury in these subjects and do not account for location or volume of injury; therefore, our adjusted analyses may not fully capture the effect of severity of head injury on ARDS or mortality outcomes. Because we studied patients with severe brain injuries that required intubation, our findings may not be generalizable to patients with less severe injuries and higher GCS scores. Furthermore, using AIS classifications to define isolated TBI has limitations. To address possible misclassification of subjects who underwent massive transfusion and therefore may have had more significant injuries beyond TBI, we performed chart review and sensitivity analyses excluding these subjects (table 3). This study did not measure TIMP-3 activity, and it is unclear if we are measuring displaced and inactive protein from tissue disruption or active protein released from tissue stores or platelet degranulation. Because the protein is measured immediately on arrival to the ED, the short time interval between injury and sampling makes it less likely that we are measuring newly synthesized protein. We are not able to comment on the origin of circulating TIMP-3 protein in the plasma. We also acknowledge that the range of TIMP-3 in this study has significant overlap between subjects who developed ARDS and those who did not, as well as subjects who survived and those who died. However the biological plausibility for the role of MMPs in the pathogenesis of complications after injury and the robust association between TIMP-3 and clinical outcomes in adjusted multivariate models support the hypothesis that TIMP-3 may be useful as one of a panel of biomarkers that could improve clinical prediction models of poor outcomes after TBI. Furthermore, we found that the incidence of ARDS and mortality rates significantly increased across TIMP-3 quartiles, suggesting that there may be a threshold above which plasma TIMP-3 levels may be particularly informative in clinical studies of postinjury complications after TBI. Further studies are needed to better understand the role of TIMP-3 in the biological pathways involved in the response to brain injury.

Our study has several strengths. Our cohort is designed for early plasma sample collection, allowing us to capture information on patients before confounding interventions and before the development of ARDS. The differences in plasma TIMP-3 levels occurred soon after injury, and measurements were unlikely to be altered by administration of fluids or blood products. The median time from EMS dispatch to ED blood draw was 34 minutes, and only 15 (8%) subjects received more than 500 mL of crystalloids prior to ED arrival. We use a rigorous adjudication protocol for ARDS, minimizing misclassification bias.^23^ Our adjusted models accounted for several measures of severity of injury. The association between TIMP-3 levels and clinical outcomes was not attenuated by measures of severity of brain injury or shock on arrival to the ED. In a multivariate model that includes clinical predictors strongly associated with the outcome, the degree of change we observed in the AUROC tests suggests that, although TIMP-3 alone is inadequate for the prediction of ARDS and death after TBI, it meaningfully improves the ability of the model to predict ARDS and death after TBI in this cohort.

## Conclusion

Our study is one of the few studies to test the association of a plasma biomarker and ARDS after isolated TBI. Our findings suggest TIMP-3 may play an important role in the biology of the systemic response to brain injury in humans. Specifically, the findings support the hypothesis that early endothelial injury and vascular permeability may be important in the pathogenesis of ARDS after TBI. Furthermore, early plasma TIMP-3 levels, in combination with clinical information and other biomarkers, could be useful in future studies to identify patients at highest risk of ARDS and death after isolated TBI.
